# A multicenter phase III study comparing Simultaneous Integrated Boost (SIB) radiotherapy concurrent and consolidated with S-1 versus SIB alone in elderly patients with esophageal and esophagogastric cancer – the 3JECROG P-01 study protocol

**DOI:** 10.1186/s12885-019-5544-1

**Published:** 2019-04-29

**Authors:** Chen Li, Xiaomin Wang, Xin Wang, Chun Han, Ping Wang, Qingsong Pang, Junqiang Chen, Xinchen Sun, Lan Wang, Wencheng Zhang, Yu Lin, Xiaolin Ge, Zongmei Zhou, Wenjie Ni, Xiao Chang, Jun Liang, Lei Deng, Wenqing Wang, Yidian Zhao, Zefen Xiao

**Affiliations:** 1 Department of Radiation Oncology, National Cancer Center/National Clinical Research Center for Cancer/Cancer Hospital, Chinese Academy of Medical Sciences and Peking Union Medical College, Beijing, 100021 China; 2 Department 4th of Radiation Oncology, Anyang Cancer Hospital, Anyang, 455000 China; 3 Department of Radiation Oncology, the Fourth Hospital of Hebei Medical University, Shijiazhuang, 050011 China; 4 Department of Radiation Oncology, Tianjin Medical University Cancer Institute and Hospital/National Clinical Research Center for Cancer, Tianjin, 300060 China; 5 Department of Radiation Oncology, Fujian Cancer Hospital/Fujian Medical University Cancer Hospital, Fuzhou, 350014 China; 6 Department of Radiation Oncology, the First Affiliated Hospital of Nanjing Medical University, Nanjing, 210029 China; 7 Department of Radiation Oncology, Cancer Hospital Chinese Academy of Medical Sciences, Shenzhen Center, Shenzhen, 518000 China

**Keywords:** Esophageal cancer, Esophagogastric junction cancer, Elderly, Concurrent chemoradiotherapy, Definitive chemoradiotherapy, Consolidated chemotherapy, Simultaneous integrated boost, Intensity modulated radiotherapy, Randomized controlled trial, S-1, Comprehensive geriatric assessment

## Abstract

**Background:**

The importance of definitive radiotherapy for elderly patients with esophageal and esophagogastric-junction cancer is pronounced. However, little is known in terms of the best way to combine radiotherapy with other treatment options. This study aims to compare the efficiency of SIB radiotherapy alone with SIB radiotherapy concurrent and consolidated with S-1 for elderly patients. Comprehensive geriatric assessment is also incorporated in the procedure of treatment.

**Methods/design:**

The study is a two arm, open, randomized multicenter Phase III trial with patients over 70 years old with stage IIA-IVB (UICC 2002, IVB only with metastasis to supraclavicular or celiac lymph nodes) squamous cell carcinoma or adenocarcinoma of esophagus or gastroesophageal junction. A total of 300 patients will be randomized using a 1:1 allocation ratio stratified by disease stage and study site. Patients allocated to the SIB arm will receive definitive SIB radiotherapy (95%PTV/PGTV 50.4Gy/59.92Gy/28f) while those randomized to SIB + S-1 arm will receive definitive SIB radiotherapy concurrent and consolidated with S-1. The primary endpoint of the trial is 1-year overall survival. Secondary objectives include progression-free survival, recurrence-free survival (local-regional and distant), disease failure pattern, toxicity profile as well as quality of life. Besides, detailed radiotherapy protocol and quality assurance procedure have been incorporated into this trial.

**Discussion:**

The proportion of elderly patients in esophageal cancer is now growing, but there is a lack of evidence in term of treatment standard for this group of patients, which is what we aim to obtain through this prospective phase III study.

**Trial registration:**

clinicaltrials.gov NCT02979691. Registered November 22, 2016.

## Background

Esophageal and esophagogastric-junction cancer (EC and EGJC) is the fourth most common cancer in China and elderly EC/EGJC patients accounts for approximately 30~40% of all cases. [1, 2]. The management of elderly patients with EC/EGJC is still challenging as their relative poor physical conditions impose great limitations on their treatment. Generally, they are considered ineligible for esophagectomy, the major component of treatment norm for patients with resectable EC/EGJC, because of the high rates of postoperative morbidity and mortality (especially those received preoperative chemoradiation) [3, 4]. A promising alternative without major toxicity is definitive chemoradiotherapy (dCRT), which is initially designed for patients with locally advanced EC/EGJC. However, the dual-drug intravenous chemotherapy regimen (fluorouracil/capecitabine + platinum drugs) recommended by National Comprehensive Cancer Network (NCCN) [5] and European Society for Medical Oncology (ESMO) [6] may bring severe acute and late toxic effects and poor compliance rate in elderly population. Thus, a modified dCRT regimen specific to the elderly is in great demand.

In this paper, we propose a prospective phase III clinical trial to improve dCRT regimen for elderly patients in three aspects: First, S-1, a single chemotherapeutic drug will be used instead of the conventional two-drug chemotherapy regimen to improve therapeutic effect while maintaining the rate of toxicities at a relatively low level. Secondly, SIB technique will be adopted to increase the dose of regions at high risk, while simultaneously reduce the dose of organs at risk (OAR). The adopted radiation dose pattern come from a prospective phase I/II trial previously conducted in our center [7]. Finally, the specialty of elderly population will be considered by integrating comprehensive geriatric assessment (CGA) into treatment procedure.

CGA, a multidisciplinary evaluation of the elderly, usually covers functional status, cognitive capacities, emotional status, comorbidities, nutritional status, social and environmental situations, and a possible geriatric syndrome. Over the past decade, the CGA has been proposed as a tool for managing elderly patients with cancer [8–14]. However, no study has ever attempted to incorporate CGA to the treatment of patients with EC/EGJC. 3JECROG P-01 trial is the first large-scale prospective phase III study to combine CGA with the treatment of elderly EC/EGJC patients to the best of our knowledge.

To summarize, this prospective, multi-center phase III clinical trial is initiated in the expectation of obtaining high-level type I evidence for the standard treatment of EC/EGJC among elderly patients.

## Methods/design

### Study design and objectives

**Fig. 1:**
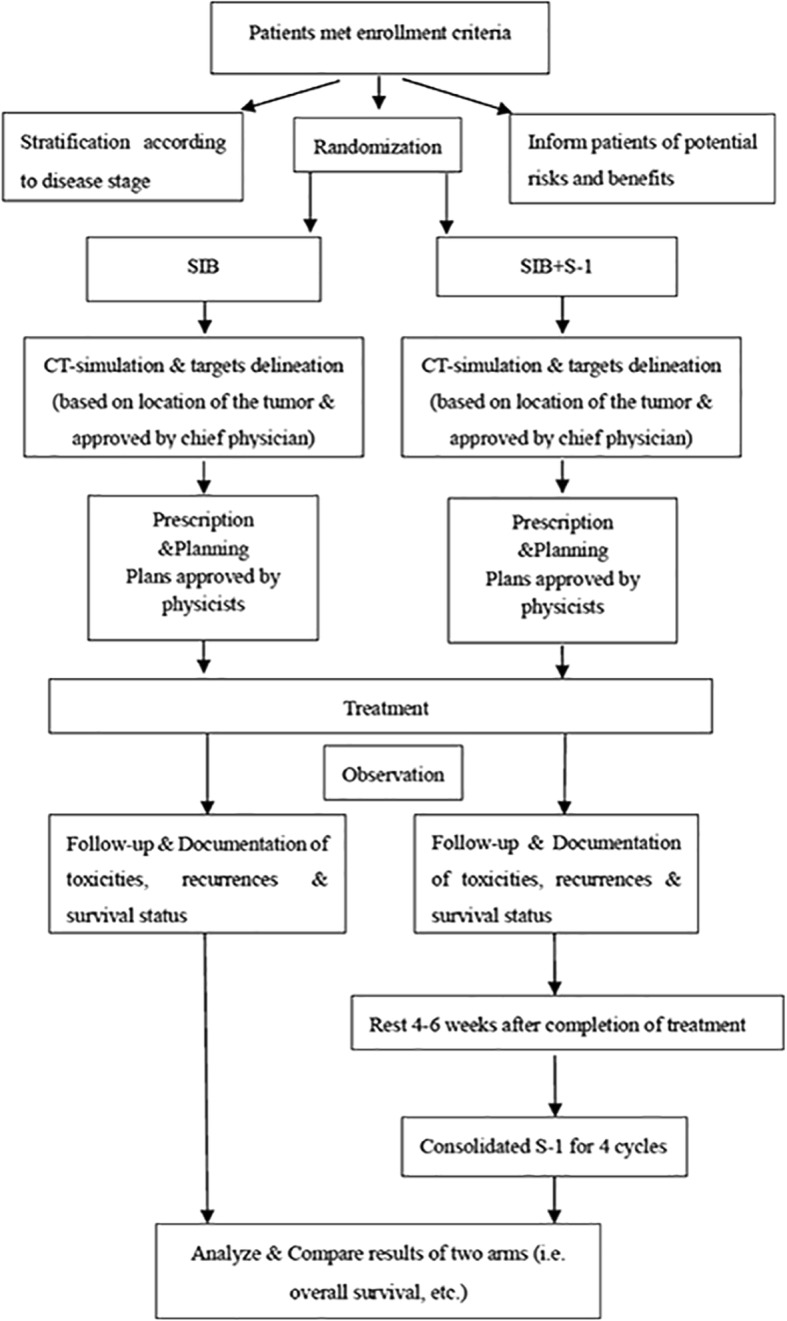
Flow chart of 3JECROG P-01

This study is an open, multicenter Phase III clinical trial. Approximately 15 participating centers throughout China are involved. The technique of SIB is adopted in this study with a dose of 50.4Gy/2.14Gy/28f to planning target volume (PTV) and 59.92Gy/2.14Gy/28f to planning gross tumor volume (PGTV). S-1 is given both concurrent with and after radiotherapy. Patients enrolled are stratified by disease stage and study site and assigned to either SIB + S-1 group or SIB group using a 1:1 allocation ratio at randomization. A flow chart giving an overview of the study design is shown in Fig. 1.

The primary objective of this trial is to compare the 1-year OS of SIB + S-1 group with SIB group. Secondary objectives include progression-free survival, recurrence-free survival (local-regional and distant), disease failure pattern, toxicity profile as well as quality of life.

The study started on February, 2017 and the duration of inclusion will be approximately 2 and a half years.

### Patient

#### Patient selection

Patients older than 70 years old with histocytologically proven stage IIA-IVB (UICC 2002, IVB only with metastasis to supraclavicular or celiac lymph nodes) inoperable squamous cell carcinoma (SCC) or adenocarcinoma (AC) of esophagus or gastroesophageal junction are eligible for recruitment. No other treatment is allowed before enrollment. For cancer of esophagogastric junction, the center of the tumor could not extend 2 cm into the gastric cardia. Patients must have adequate hematological, renal and hepatic functions defined as: neutrophils ≥3.5 × 10^9^/L, granulocytes ≥1.5 × 10^9^/L, platelets ≥100 × 10^9^/L, urea nitrogen (BUN) ≤ 1.0 × upper normal limit (UNL), creatinine (Cr) ≤ 1.0 × upper normal limit (UNL), alanine aminotransferase (ALT), aspartate aminotransferase (AST) ≤1.5× UNL; alkaline phosphatase (ALP) ≤ 1.5× UNL; total bilirubin ≤UNL. General condition of enrolled patients must be acceptable (i.e. Karnofsky Performance Status≥70 or Eastern Cooperative Oncology Group performance status ≤1, Charlson Comorbidity Index≤3).

Exclusion criteria include prior malignancies (other than curable non-melanoma skin cancer or in situ cervical cancer) within 5 years, distant lymph node (other than metastasis to supraclavicular or celiac lymph nodes) or visceral metastasis (including metastasis to bone, lung, liver, pleura or peritoneum, etc.), uncontrolled infection such as tuberculosis or hepatitis, uncontrolled hypertension or diabetes, severe comorbidities such as myocardial infarction, cerebral embolism or serious arrhythmia within 6 months, obvious sign of esophageal perforation or deep ulcer affirmed by barium esophagram.

#### CGA

As mentioned before, due to the specialty of elderly population, enrolled patients will receive a comprehensive assessment called CGA (including social support, general status, body function, nutritional status, mental health, cognitive ability and so forth) both before and after treatment. Detailed procedures of CGA are listed in Table 1.

**Table 1:** Domains and measures accessed by CGA before and after treatment

Domain with measure	No. of items	Range of score
Quality of life and specific module for EC		
EORTC QLQ-C30 (Version 3.0) [34]	30	30–126
QLQ-OES-18 [35]	18	18–72
Functional status		
ECOG performance status [36]		0–5
Karnofsky performance status [37]		0–100
Activities of daily life (Barthel index) [38]	10	0–100
Instrumental activities of daily life (Lawton’s) [39]	8	0–5/8
Comorbidity		
Charlson comorbidity index [40]	19	0–37
Cognitive status		
Mini Mental State Examination [41]	11	0–30
Psychological status		
Geriatric depression scale-5 items [42]	5	0–5
Nutritional status		
Body mass index [43]		
Mini-nutritional assessment [44]	18	0–100
Social support		
Medical Outcomes Study - social support survey [45]	20	20–100

*CGA* comprehensive geriatric assessment, *EC* esophageal cancer, *ECOG* Eastern Cooperative Oncology Group, *EORTC* The European Organization for Research and Treatment of Cancer, *MOS* Medical outcomes study

### Radiotherapy

For patients with lower-thoracic EC or EGJC, a series of pre-treatment procedure will be adopted to minimize the effect of stomach-volume-variation during treatment delivery. Patients should be fasted for at least 4 h and then drink 200-300 ml semiliquid 15 min before CT simulation and daily irradiation. Supine position is performed with both arms straight beside the body. Head and neck hood is recommended for patients with cervical or upper-thoracic EC, while body film is used for middle, lower-thoracic EC and EGJC.

The Gross Tumor Volume (GTV-T) is defined as the primary tumor. The GTV-T will be determined using all available resources (physical examination, upper gastrointestinal contrast, endoscopy, EUS, CT-thorax/abdomen, MRI-thorax/abdomen, PET-CT, etc.).

The metastatic regional nodes (GTV-N) is defined as any lymph node diagnosed as or highly-suspected as metastatic.

**Fig. 2:**
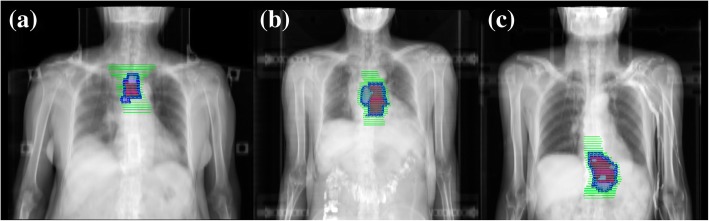
Targets contouring of (**a**) upper thoracic esophagus (Ut); (**b**) middle thoracic esophagus (Mt); (**c**) lower thoracic esophagus (Lt). Red area indicates GTV-T, grey area includes GTV-N, blue area outlines PGTV and green area outlines PTV

As for the contouring of Clinical Target Volume (CTV), Involved-field radiotherapy (IFRT) is adopted. CTV consists of GTV-T plus a 0.6 to 0.8 cm circumferential margin, a 3 cm craniocaudal margin and GTV-N plus a 0.5 cm margin in all directions. For patients whose highest/lowest metastatic lymph nodes not exceeding 3 cm from the primary tumor, the upper/lower border of the is 3 cm superior/inferior to the primary tumor. For patients whose highest/lowest metastatic lymph nodes exceeding 3 cm from primary tumor, the upper/lower boundary of CTV is 0.5 cm superior/inferior to the furthest metastatic lymph node. No prophylactic irradiation is given to lymph node drainage regions. The PGTV is created by expanding GTV-T by 1.0 cm craniocaudally and 0.5 cm radially and GTV-N by a uniform 0.5 cm margin, and the PTV is derived from CTV plus a uniform 0.5 cm margin. The typical contouring of targets for tumor in different locations are depicted in Fig. 2 respectively.

The radiotherapy technique of SIB is performed 5 times a week. For thoracic esophagus, prescription dose is 95% PTV/PGTV 50.40Gy/59.92Gy/28f. For EGJC, prescription dose is 95% PTV/ PGTV 45.0Gy/53.5Gy/25f.

Both lungs, heart, spinal cord and spinal cord PRV should be contoured on the simulation images. For tumor of lower-thoracic EC and EGJC, physicians should also delineate stomach, liver, both kidneys and bowels (including small intestine and colon) as OARs. Bowels and spinal cord should be contoured 2 cm superior and inferior to PTV. OARs such as lungs, heart, stomach, kidneys and liver should be delineated from their upper border to their lower end. The volume of lung tissue receiving 20Gy or more should not exceed 28% of the total lung volume (V20 **<** 28%). The mean dose of lung tissue should be lower than 16Gy (Dmean lung **<**16Gy). Other dose constraints to OARs include: V40 heart **<** 30%, V30 heart **<** 40%, V40 stomach **<** 40%, Dmax stomach **<** 55-60Gy, V40 small intestine **<** 40%, Dmax small intestine **<**55Gy, V30 liver **<** 30%, V20 kidney **<** 30% and Dmax spinal cord PRV **<** 45Gy.

### Chemotherapy

S-1 will be orally taken twice daily (or through nasal feeding tube for patients with enteral nutrition) within half an hour after meals during treatment days. It should not be taken during weekends or whenever radiotherapy is interrupted or stopped. The specific dosage for each patient is calculated according to body surface area in Table 2:

**Table 2:** Relationship between body surface area and dose of S-1

Body surface area (m^2^)	Initial dose
<1.25	40 mg, bid
≥1.25~<1.50	50 mg, bid
≥1.5	60 mg, bid

**Fig. 3:**
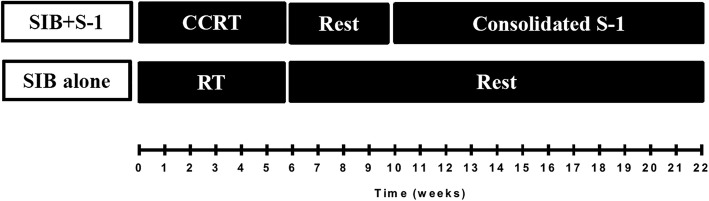
Treatment schedule for both arms CCRT: Concurrent Chemoradiotherapy; RT: Radiotherapy

Generally, 4–8 weeks are needed for patients to recover after completion of SIB + S-1 (status of food intake, physical capacity, biochemical tests, etc.). Four cycles of S-1 will be given to those eligible for consolidation chemotherapy. Treatment schedule for patients in both arms is shown in Fig. 3. The daily-dose of consolidated S-1 is the same as that of concurrent phase, but it will be administered for the first 2 weeks on each 21-days cycle. Blood routine should be monitored every week and hepatic and renal functions every cycle during chemotherapy.

### Toxicity and adverse events

All treatment-related toxicities and adverse events should be graded according to the toxicity criteria of the Radiation Therapy Oncology Group (RTOG) and Common Terminology Criteria of Adverse Events (CTCAE) version 4.0 and recorded on patients’ case report form (CRF) in detail. Serious Adverse Events (SAE) should be reported to the institutional ethical review committee in 24 h and dealt with properly.

S-1 should be terminated in case of Grade 2 anemia, thrombocytopenia, hepatic or renal dysfunction, Grade 4 leukopenia/neutropenia, Grade 3 radiation esophagitis and other non-homological Grade 3 toxicities. If adverse events de-grade to Grade 0–1 within 1 week of drug withdrawal, patient can re-take S-1 at original dose, otherwise chemotherapy should be stopped henceforward. If Grade 3/4 radiation pneumonitis developed, both radiotherapy and oral intake of S-1 should be terminated. Consolidation of S-1 will be re-assessed within 4–8 weeks after radiotherapy whichever grade of toxicities developed during dCRT.

### Follow-up

Tumor regression should be evaluated according to RECIST Version 1.1 within 1–2 month after completion of treatment and rates of cCR and cPR should be documented. All patients should be followed up for at least 5 years after completion of the protocol and the time interval is every 3 months for the first 2 years, every 6 months for 3–5 years and once a year after 5 years.

Every follow-up should include: a. History-taking: symptoms of cough, fever, hoarseness, dysphagia or chest tightness, etc. b. Blood routine, basic metabolic panel, tumor markers, etc. c. Contrast enhanced CT of neck, thorax and abdomen, ultrasound of neck and abdomen, upper gastrointestinal contrast, bone scan (in case of bone pain or abnormally-elevated alkaline phosphatase), CT or MRI of brain (in case of any symptoms related to central nervous system), etc. d. Documentation of patients’ status of survival, disease progression, subsequent treatment, nutrition, life quality and late toxic effects, etc.

### Statistical analysis & sample size considerations

We assume that an estimated difference in 1-y OS of 65% (SIB arm) versus 75% (SIB + S-1 arm) would justify applying this regimen in the future. Assuming a one-sided significance level of 0.05, a power of 0.80, and 10% of lost in each arm, a total of 300 patients would be needed in this trial.

Estimates of median OS will be based on the Kaplan-Meier method and log-rank tests will be used to determine the significance. Cox regression analysis will be conducted to identify prognostic factors for survival benefit, which will be used in adjusted analyses of the treatment effect.

### Ethics

The charge doctor should inform enrolled patients of the background of both treatment options, especially known efficiency and toxicities. It must be emphasized that the patient is allowed to refuse the treatment either before or during the study. Before enrollment, the patient’s written consent should be obtained. The principal investigator (PI) will ensure that this study will be carried out in agreement with the “Declaration of Helsinki, Tokyo, Venice” or the laws and regulations of the country, whichever provides greater protection of the individual. The study has been approved by the institutional ethical review committee.

### Quality assurance

A strict coordination and monitoring system has been constructed for this trial. First of all, a Radiotherapy Trials Quality Assurance (RTTQA) team consisted of physicians, dosimetrists, medical physicist and research fellows was built before the start-up of enrollment. Moreover, every participated branch-center should nominate at least one physician responsible for patient recruitment, data collection and online fill-out of CRF of their center. The RTTQA team will also name at least one censor to evaluate the quality of data collected from all branch centers and the censor is also in charge of keeping sound communication with physicians in charge from all participated centers.

Great effort has been put on assurance of treatment-quality and equality of all involved centers. An EC case was selected as an example by RTTQA team and distributed to all branch centers at the beginning of the study together with a brief clinical summary and CT imaging data of the selected case. All participated centers should send the case back to RTTQA team after completion of targets-delineation and RTTQA team would assess all collected cases for major and minor deviations. This is what we call the first round of Collection of Targets-Delineation (CTD). After that, the RTTQA team distributed a detailed protocol for targets-delineation to all branch-centers and let physicians in charge to contour targets again on the same sample case [15]. The countered case was collected again and this is what we call the second round of CTD. The second round of radiotherapy plans were also viewed thoroughly by the RTTQA team. It was proved that both quality and equality of the radiotherapy treatment improved significantly after distribution of the treatment protocol. This procedure assures that all centers and investigators have had a ‘Pass’ for the planned test case before entering patients into the trial. Censors from the RTTQA team will also randomly inspect the quality of treatment from branch centers during the study, on what occasion physicians in charge are required to provide a dataset of images, structures, radiotherapy plans and doses to the censor.

## Discussion

In the proposed regimen, we replace the conventional two-drug chemotherapy regimen (fluorouracil/capecitabine + platinum drugs) by S-1, a single chemotherapeutic drug taken orally. The major consideration is to avoid the high toxic effects of the dual-drug chemotherapeutic regimen. In the study of RTOG 85–01 and INT-0123 [16, 17], although the enrolled population were mostly non-elderly patients under the age of 70, dual-drug based chemoradiotherapy still brought serious adverse reactions. The rates of Grade 3–4 hematological toxicities were 48% vs 3% and that of gastrointestinal reaction were 33% vs 18%, respectively. For elderly patients, worse results can be expected. Thus, the chemotherapeutic regimen from RTOG 8501 is generally modified for elderly patients in several small-sample prospective and retrospective studies. Generally, the toxicities remain at high levels even after these modifications if dual-drug chemotherapeutic regimen is adopted [18, 19]. On the other hand, single-drug low-toxic chemotherapeutic regimen seems to be a promising treatment mode for elderly patients as they can reduce the toxic effects significantly. A prospective study evaluated the effect of radiation concurrent with single-drug agent (cisplatin/oxaliplatin) among patients older than 75 years. With a 53% cCR and 22% 3-y OS, only 3% patients had grade 3/4 hematological reactions during treatment [20]. In our study, S-1 is adopted as single concurrent and consolidated drug for its high efficiency and low toxicity. As a new generation of 5FU derivatives, S-1 showed better therapeutic effect than 5FU in some malignancies such as small cell lung cancer and pancreatic cancer [21]. It also led to significant lower rates of Grade 3/4 neutropenia (HR = 0.33, 95%CI 0.25–0.44) among patients with metastatic gastric cancer compared to 5FU [22]. Besides, S-1, a capsule preparation, provides a more convenient way of administration for elderly patients compared with continuous infusion of 5FU.

Another highlight of our study is the use of SIB. Although the dose recommended by most guidelines [5, 6] is 50.4Gy, the local control rate (LCR) under 50.4Gy is unsatisfying [23] and higher doses may be needed for high-risk areas. Besides, some retrospective study [24] have shown that in the subgroup of SCC, patients received high-dose irradiation (≥60Gy) had better OS and LCR than those who only received conventional dose (50.4Gy). Moreover, dosimetry studies have shown that for EC/EGJC, with the help of SIB, one can successfully increase the dose of boost areas without increasing the irradiation of OARs [25, 26]. Thus this technique has been used more and more widely and most institutions have given boost areas a dose≥60Gy. [27–30]. The dose pattern adopted in this trial (95%PGTV/PTV 59.92Gy/50.40Gy/28f, EQD2 = 60.62Gy) is derived from a prospective Phase I/II trial previously conducted in our center [7]. According to that study, the use of SIB in dCRT patients is safe and feasible at 59.92Gy/50.40Gy/28f and the survival results are satisfying (1-y OS and local failure-free survival were 76.9 and 78.8% respectively). Therefore, we apply that dose model in this phase III study. To the best of our knowledge, this is the first large-scale prospective randomized study using SIB in the treatment of EC/EGJC.

Finally, 3JECROG P-01 is also the first multicenter prospective trial to incorporate CGA, a multidimensional diagnostic process investigating medical, psychosocial and physiological functions of the elderly, to the treatment of patients with EC/EGJC. It has been confirmed that the application of CGA in elderly patients with cancers can lower the rate of treatment-related complications, improve their QoL and body function and reduce the risk of hospitalization, etc. [31] Thus, CGA has been increasingly involved in the treatment of aged cancer patients worldwide [32, 33]. In this study, the relationship between CGA and survival status, the incidence of side effects and QoL in elderly patients will be analyze to explore the value of CGA in guiding the individualized treatment for elderly patients with EC/EGJC.

## Abbreviations

AC: Adenocarcinoma
ALP: Alkaline Phosphatase
ALT: Alanine Aminotransferase
AST: Aspartate Aminotransferase
BUN: Blood Urea Nitrogen
cCR: clinical Complete response
CCRT: Concurrent chemoradiotherapy
CGA: Comprehensive Geriatric Assessment
cPR: clinical Partial response
Cr: Creatinine
CRF: Case Report Form
cRR: clinical Response Rate
CT: Computed Tomography
CTCAE: Common Terminology Criteria for Adverse Events
CTD: Collection of Targets-Delineation
CTV: Clinical target volume
dCRT: definitive Concurrent chemoradiotherapy
EC: Esophageal cancer
ECOG: Eastern Cooperative Oncology Group
EGJC: Esophagogastric junction cancer
EQD2: Equivalent Dose in 2 Gy/f
ESMO: European Society for Medical Oncology
GTV-N: Metastatic regional nodes
GTV-T: Gross Tumor Volume
IDMC: Independent Data Monitoring Committee
ITT: Intention to treat
LCR: Local Control Rate
Lt: Lower thoracic
MRI: Magnetic Resonance Imaging
Mt: Middle thoracic
MTD: Maximum tolerated dose
NCCN: National Comprehensive Cancer Network
OAR: Organs at risk
OS: Overall Survival
PGTV: Planning Gross Tumor Volume
PP Per: Protocol
PTV: Planning target volume
RECIST: Response Evaluation Criteria In Solid Tumors
RT: Radiotherapy
RTOG: Radiation Therapy Oncology Group
RTTQA: Radiotherapy Trials Quality Assurance
S-1: Tegafur,Gimeracil and Oteracil Potassium Capsules
SAE: Serious adverse event
SCC: Squamous cell carcinoma
SCLC: Small cell lung cancer
SIB: Simultaneous integrated boost
UNL: Upper Normal Limit
Ut: Upper thoracic
