# Correction to: A multicenter phase III study comparing Simultaneous Integrated Boost (SIB) radiotherapy concurrent and consolidated with S-1 versus SIB alone in elderly patients with esophageal and esophagogastric cancer – the 3JECROG P-01 study protocol

**DOI:** 10.1186/s12885-019-5699-9

**Published:** 2019-05-23

**Authors:** Chen Li, Xiaomin Wang, Xin Wang, Chun Han, Ping Wang, Qingsong Pang, Junqiang Chen, Xinchen Sun, Lan Wang, Wencheng Zhang, Yu Lin, Xiaolin Ge, Zongmei Zhou, Wenjie Ni, Xiao Chang, Jun Liang, Lei Deng, Wenqing Wang, Yidian Zhao, Zefen Xiao

**Affiliations:** 1 Department of Radiation Oncology, National Cancer Center/National Clinical Research Center for Cancer/Cancer Hospital, Chinese Academy of Medical Sciences and Peking Union Medical College, Beijing, 100021 China; 2 Department 4th of Radiation Oncology, Anyang Cancer Hospital, Anyang, 455000 China; 3 Department of Radiation Oncology, the Fourth Hospital of Hebei Medical University, Shijiazhuang, 050011 China; 4 Department of Radiation Oncology, Tianjin Medical University Cancer Institute and Hospital/ National Clinical Research Center for Cancer, Tianjin, 300060 China; 5 Department of Radiation Oncology, Fujian Cancer Hospital/Fujian Medical Li et al. BMC Cancer (2019) 19:397 Page 7 of 9 University Cancer Hospital, Fuzhou, 350014 China; 6 Department of Radiation Oncology, the First Affiliated Hospital of Nanjing Medical University, Nanjing, 210029 China; 7 Department of Radiation Oncology, Cancer Hospital Chinese Academy of Medical Sciences, Shenzhen Center, Shenzhen, 518000 China

**Correction to: BMC Cancer, 2019; 19:397**

**https://doi.org/10.1186/s12885-019-5544-1**

Following publication of the original article [1], the authors reported that an error in the Methods/Design, Study design and objectives section (third sentence). The dose of “50.4Gy/2.14Gy/28f” is incorrect and should be “50.4Gy/1.80Gy/28f”.

The sentence should read: The technique of SIB is adopted in this study with a dose of 50.4Gy/1.80Gy/28f to planning target volume (PTV) and 59.92Gy/2.14Gy/28f to planning gross tumor volume (PGTV).

The authors apologise for the inconvenience caused.

## References

[1] Li (2019). A multicenter phase III study comparing Simultaneous Integrated Boost (SIB) radiotherapy concurrent and consolidated with S-1 versus SIB alone in elderly patients with esophageal and esophagogastric cancer – the 3JECROG P-01 study protocol. BMC Cancer.

